# Structural basis of checkpoint blockade by monoclonal antibodies in cancer immunotherapy

**DOI:** 10.1038/ncomms13354

**Published:** 2016-10-31

**Authors:** Ju Yeon Lee, Hyun Tae Lee, Woori Shin, Jongseok Chae, Jaemo Choi, Sung Hyun Kim, Heejin Lim, Tae Won Heo, Kyeong Young Park, Yeon Ji Lee, Seong Eon Ryu, Ji Young Son, Jee Un Lee, Yong-Seok Heo

**Affiliations:** 1 Department of Chemistry, Konkuk University, 120 Neungdong-ro, Gwangjin-gu, Seoul 05029, Republic of Korea; 2 Department of Bio Engineering, Hanyang University, 222 Wangsimni-ro, Seongdong-gu, Seoul 04763, Republic of Korea

## Abstract

Cancer cells express tumour-specific antigens derived via genetic and epigenetic alterations, which may be targeted by T-cell-mediated immune responses. However, cancer cells can avoid immune surveillance by suppressing immunity through activation of specific inhibitory signalling pathways, referred to as immune checkpoints. In recent years, the blockade of checkpoint molecules such as PD-1, PD-L1 and CTLA-4, with monoclonal antibodies has enabled the development of breakthrough therapies in oncology, and four therapeutic antibodies targeting these checkpoint molecules have been approved by the FDA for the treatment of several types of cancer. Here, we report the crystal structures of checkpoint molecules in complex with the Fab fragments of therapeutic antibodies, including PD-1/pembrolizumab, PD-1/nivolumab, PD-L1/BMS-936559 and CTLA-4/tremelimumab. These complex structures elucidate the precise epitopes of the antibodies and the molecular mechanisms underlying checkpoint blockade, providing useful information for the improvement of monoclonal antibodies capable of attenuating checkpoint signalling for the treatment of cancer.

## Abstract (web summary)

Immunotherapy is offering patients with cancer new therapy options. Here, the authors report on the crystal structures of some of these therapies bound to their targets.

As the immune system plays an important role in controlling cancer, utilizing the immune system to eliminate cancer holds great potential. Although various immunotherapeutic approaches have been shown to enhance the immune system's ability to modulate cancer, therapeutic antibodies that target regulatory pathways in T-cells to enhance antitumor immune responses, have attracted significant recent attention.

T-cell-mediated immune responses are triggered through the recognition of antigenic peptide/HLA complexes on the surface of antigen presenting cells (APCs) by T-cell receptors and are tightly regulated by antigen-independent co-receptor signals, either costimulatory or coinhibitory, providing the optimal balance between immune responses to antigens and maintenance of self-tolerance under normal physiological conditions^1^,^2^,^3^. Costimulatory signals are required to enhance and sustain the function of T-cells, the most important of which is provided by the interaction of CD28, a co-receptor on T-cells, with its ligands B7-1 and B7-2 on APCs (refs 4, 5). In contrast, the binding of the same B7 ligands to cytotoxic T lymphocyte-associated antigen 4 (CTLA-4), a CD28 homologue with 31% sequence identity, delivers coinhibitory signals for down-regulation of immune responses^6^. Programmed death-1 (PD-1) is also an antigen-independent co-receptor and plays a pivotal role in modulating immune responses^7^. The interaction of PD-1 with its ligands PD-L1 and PD-L2 on APCs induces inhibitory signals reducing T-cell activity^8^,^9^. Although both CTLA-4 and PD-1 are coinhibitory receptors, each plays a non-redundant role in the negative regulation of immune responses. While engagement of CTLA-4 by B7 ligands attenuates the early activation of naïve and memory T-cells, PD-1 modulates the function of T-cells later in peripheral tissues via interaction with PD-L1 and PD-L2 (ref. 10).

As cancer cells harbour genetic and epigenetic modifications, tumour-specific antigens are presented on the cancer cell surface and can be recognized by T-cells, therefore causing immune responses^11^,^12^,^13^,^14^. However, cancer cells can also evade immunological recognition and destruction through the activation of coinhibitory signalling by overproduction of immune checkpoint proteins such as PD-1 and CTLA-4 on immune effector cells and PD-L1 on cancer cells^15^,^16^,^17^. Furthermore, expression of PD-L1 on cancer cells can directly lead to the death of antigen-specific effector T-cells expressing PD-1 (ref. 18). In an inflamed tumour microenvironment, engagement of PD-1 or CTLA-4 can self-limit the antitumor immune responses and permit cancer cells to proliferate unrestrained. Advances in the understanding of the molecular mechanisms underlying the ability of cancer cells to suppress immune surveillance have devised strategies to overcome cancer-induced immune tolerance, thereby protecting the host from tumour progression. Blockade of the ligand-receptor interaction of these immune checkpoint molecules can directly enhance the function of T-cells, which represents a critical paradigm shift whereby checkpoint blockade aims at disinhibition of the activity of T-cells compared with the previous immuno-oncology concept, whereby cancer vaccines and cytokine therapies aimed at *de novo* activation of immune responses.

Monoclonal antibodies blocking immune checkpoints have demonstrated unprecedented therapeutic benefits in clinical trials and provided a major breakthrough in oncology^19^,^20^,^21^,^22^,^23^. While targeted therapies elicit transient clinical responses because of acquisition of cancer drug resistance usually occurring within months after an initial response, the clinical responses of checkpoint blockade therapies are often durable, with some patients free from cancer progression for many years^19^,^24^,^25^. The US Food and Drug Administration (FDA) has approved monoclonal antibodies that block CTLA-4 (ipilimumab), PD-1 (pembrolizumab and nivolumab), and PD-L1 (atezolizumab) for the treatment of melanoma, non-small-cell lung cancer, renal cell carcinoma, Hodgkin lymphoma and bladder cancer. As a number of clinical trials for antibodies targeting immune checkpoints are ongoing, additional approvals are expected for other antibodies and expanded indications in the near future^26^.

There have been a number of structural studies for immune checkpoint molecules. The structures of the extracellular portion of CTLA-4 revealed an unusual mode of CTLA-4 dimerization that places the B7 binding sites distal to the dimerization interface^27^. The crystal structures of CTLA-4 in complex with its B7 ligands showed the formation of a unique alternating network of bivalent CTLA-4 and B7-1/2 dimers, providing a structural basis for formation of unusually stable signalling complexes to regulate T-cell responsiveness within the immunological synapse^28^,^29^. The structures of murine PD-1 in complex with human PD-L1, murine PD-1 in complex with murine PD-L2, and human PD-1 in complex with human PD-L1 have established the structural foundations of the interaction of PD-1 with its ligands^30^,^31^,^32^,^33^. These structural studies for checkpoint molecules and their binding complexes have provided invaluable information for understanding the molecular mechanism of coinhibitory signals of T-cells through the communication of checkpoint molecules.

As the exact epitope and mechanism are crucial elements for antibody drugs, we report the crystal structures of checkpoint molecules in complex with the Fab fragments of therapeutic antibodies approved by the FDA or currently undergoing clinical trials, and reveal the binding modes of the complexes and the conformational changes induced by the antibody binding, thereby providing a structural basis of checkpoint blockade by monoclonal antibodies for the treatment of cancer.

## Results

### Crystal structures of PD-1 in complex with anti-PD-1 drugs

**Figure 1:**
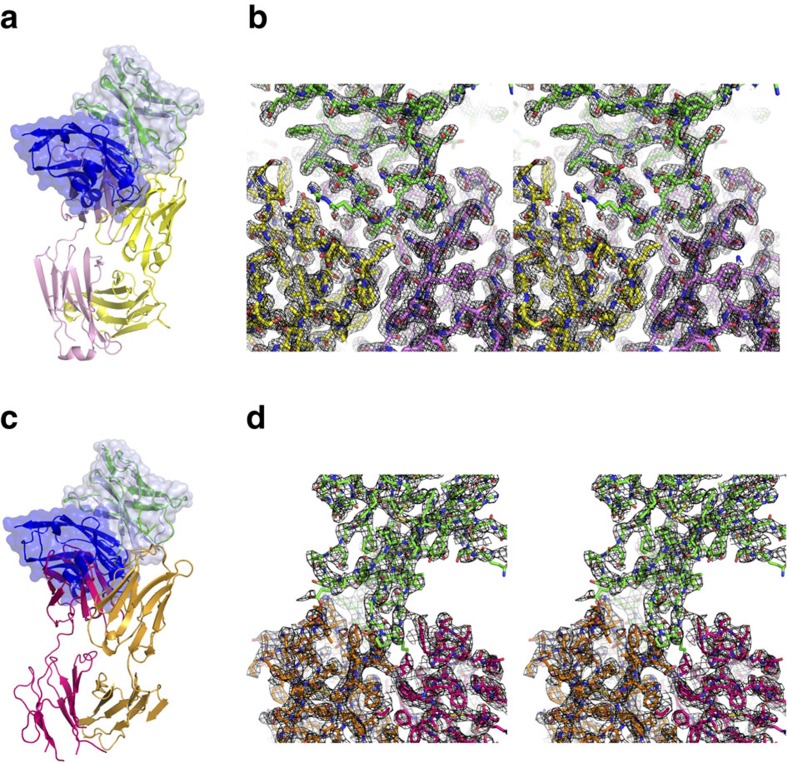
Crystal structures of PD-1 in complex with anti-PD-1 drugs. (**a**) Ribbon representation of the complex structure of PD-1/pembrolizumab Fab fragment. The heavy and light chains of pembrolizumab are coloured pink and yellow, respectively. PD-1 in the complex is coloured green. (**b**) Stereoview of the 2fo-fc composite omit map (1.2 σ contour level) at the interface between PD-1 (green) and pembrolizumab (heavy chain: pink, light chain: yellow) calculate at 2.0 Å resolution. (**c**) Ribbon representation of the complex structure of PD-1/nivolumab Fab fragment. The heavy and light chains of nivolumab are coloured orange and purple, respectively. PD-1 in the complex is coloured green. (**d**) Stereoview of the 2fo-fc composite omit map (1.2 σ contour level) at the interface between PD-1 (green) and nivolumab (heavy chain: orange, light chain: purple) calculated at 3.3 Å resolution. In **a** and **c,** the PD-1/PD-L1 complex (PDB code 4zqk) was superimposed onto the PD-1 molecule in the PD-1/anti-PD-1 complexes with mixed ribbon/surface representation. PD-1 and PD-L1 in the PD-1/PD-L1 complex are coloured pale blue and blue, respectively.

**Figure 2:**
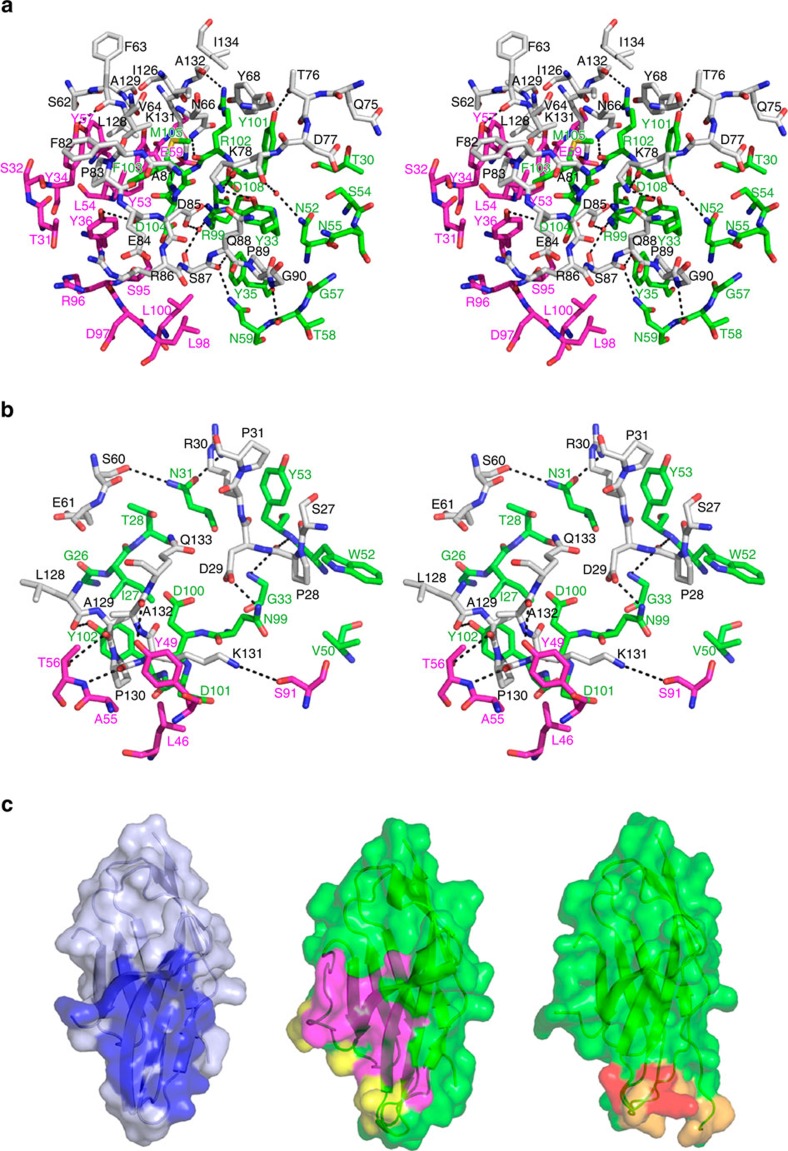
Interactions between PD-1 and anti-PD-1 drugs. (**a**) Stereoview of the detailed PD-1/pembrolizumab interface. (**b**) Stereoview of the detailed PD-1/nivolumab interface. In **a** and **b**, the carbon atoms of PD-1 and heavy and light chain of anti-PD-1 are coloured grey, green, and purple, respectively. Hydrogen bonds and a salt bridge are indicated with dashed lines. (**c**) Surface representations of the PD-1 molecules in the complex structures of PD-1/PD-L1 (left), PD-1/pembrolizumab (centre) and PD-1/nivolumab (right). The surface of PD-1 in PD-1/PD-L1 is coloured pale blue and the PD-L1 binding site on the surface of PD-1 is coloured blue. The surfaces of PD-1 in PD-1/anti-PD-1 are coloured green. The epitope regions for the heavy and light chains of pembrolizumab are coloured purple and yellow, respectively. The epitope regions for the heavy and light chains of nivolumab are colored orange and red, respectively.

Pembrolizumab (trade name KEYTRUDA) is a humanized IgG4 antibody that blocks PD-1 (ref. 26). The FDA approved pembrolizumab for the treatment of advanced melanoma in September 2014 and metastatic non-small-cell lung cancer (NSCLC) in October 2015. The PD-1/pembrolizumab Fab fragment complex was crystallized and its structure was determined and refined to a resolution of 2.0 Å (Fig. 1a,b). The pembrolizumab epitope of PD-1 consists of numerous discontinuous segments (Fig. 2a). The formation of hydrogen bonds between PD-1 and pembrolizumab involves side chain atoms of _PD1_N66, _PD1_T76, _PD1_K78, _PD1_S87 and _PD1_K131 and main chain atoms of _PD1_F63, _PD1_E84, _PD1_S87, _PD1_G90 and _PD1_A132, whereas a salt bridge is formed between _PD1_D85 and _heavy_R99. In addition, the main chain atom of _PD1_K78 and the side chain atoms of _PD1_S87 and _PD1_D85 participate in water-mediated interactions with _heavy_N52 and _heavy_R99. The residues of PD-1 involved in van der Waals contact with pembrolizumab are _PD1_S62, _PD1_V64, _PD1_Y68, _PD1_Q75, _PD1_D77, _PD1_A81, _PD1_F82, _PD1_P83, _PD1_R86, _PD1_Q88, _PD1_P89, _PD1_G90, _PD1_I126, _PD1_L128, _PD1_A129, and _PD1_I134. The paratope of pembrolizumab consists of _heavy_T30, _heavy_Y33, and _heavy_Y35 of HCDR1; _heavy_N52, _heavy_S54, _heavy_N55, _heavy_G57, _heavy_T58, and _heavy_N59 of HCDR2; _heavy_R99, _heavy_Y101, _heavy_R102, _heavy_F103, _heavy_D104, _heavy_M105, and _heavy_D108 of HCDR3; _light_T31, _light_S32, _light_Y34, and _light_Y36 of LCDR1; _light_Y53, _light_L54, _light_Y57, and _light_E59 of LCDR2; and _light_S95, _light_R96, _light_D97, _light_L98 and _light_L100 of LCDR3.

Nivolumab (trade name OPDIVO) is a fully human IgG4 that blocks PD-1 (ref. 26). The FDA approved nivolumab for the treatment of melanoma in December 2014, NSCLC in March 2015, renal cell carcinoma in November 2015, and Hodgkin lymphoma in May 2016. The PD-1/nivolumab Fab complex and free nivolumab Fab fragment were crystallized and their structures were determined and refined to a resolution of 3.3 and 1.9 Å, respectively (Fig. 1c,d). In the complex structure, the formation of hydrogen bonds between PD-1 and nivolumab involves side chain atoms of _PD1_D29, _PD1_R30, _PD1_S60 and _PD1_K131 and main chain atoms of _PD1_P28, _PD1_L128, _PD1_A129, _PD1_P130, and _PD1_A132. The PD-1 residues involved in van der Waals contact with nivolumab are _PD1_S27, _PD1_P28, _PD1_P31, _PD1_E61, _PD1_A129, _PD1_P130, _PD1_K131, _PD1_A132 and _PD1_Q133. The residues of nivolumab involved in the interaction with PD-1 are _heavy_G26, _heavy_I27, _heavy_N31 and _heavy_G33 of HCDR1; _heavy_V50, _heavy_W52 and _heavy_Y53 of HCDR2; _heavy_N99, _heavy_D100, _heavy_D101 and _heavy_Y102 of HCDR3; _light_L46, _light_A55 and _light_T56 of LCDR2; and _light_S91 of LCDR3 (Fig. 2b).

**Figure 3:**
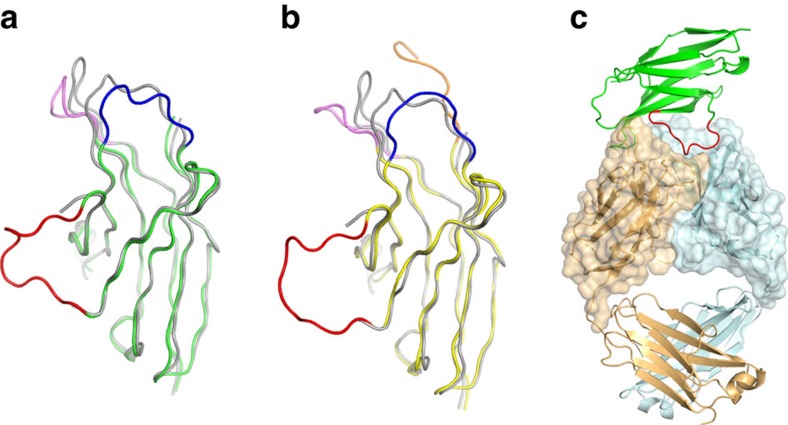
Conformational changes in the loops of PD-1 induced by the binding of anti-PD-1 antibodies. (**a**) Superposition of PD-1 molecules extracted from the complexes of PD-1/PD-L1 (grey, PDB code 4zqk) and PD-1/pembrolizumab (green), yielding r.m.s. deviation of 1.26 Å. (**b**) Superposition of PD-1 molecules extracted from the complexes of PD-1/PD-L1 (grey) and PD-1/nivolumab (yellow), yielding r.m.s. deviation of 1.38 Å. In **a** and **b**, the BC, C′D, and FG loops of the PD-1 molecule in PD-1/anti-PD-1 are coloured blue, red and violet, respectively. In **b**, the N-terminal region (residue 27-33) of the PD-1 molecule in PD-1/nivolumab is coloured orange. This N-terminal region in PD-1/PD-L1 and PD-1/pembrolizumab and the C′D loop in PD-1/PD-L1 are not shown in the structures due to the lack of interaction. (**c**) Interaction of the C'D loop with pembrolizumab with ribbon representation of PD-1 (green) in complex with pembrolizumab Fab (heavy chain: pale cyan, light chain: pale orange). Surface of the Fv region of pembrolizumab is also represented. The C′D loop of PD-1 is coloured red.

The total buried surface areas of the PD-1/pembrolizumab and PD-1/nivolumab complexes are 2,126 Å^2^ and 1,487 Å^2^, respectively, compared with 1,970 Å^2^ for PD-1/PD-L1 (ref. 30). The epitopes of the two antibodies directly occupy part of the PD-L1 binding site (Fig. 2c). In addition, the binding of PD-1 with pembrolizumab or nivolumab induces optimal conformational changes in the BC loop (residues 57–63) and the FG loop (residues 127–134) of PD-1, which are incompatible with PD-L1 binding as they also interact with PD-L1 in distinct conformations^30^ (Fig. 3a,b). The C′D loop (residues 81–90) was shown to be highly flexible in the NMR structure of free PD-1 (ref. 31), and disordered in the crystal structure of the PD-1/PD-L1 complex as a result of the lack of interaction^30^,^32^,^33^. However, most of the residues of the C′D loop in the PD-1/pembrolizumab complex are involved in the interactions with pemrolizumab with a clear electron density around them, implying that the C′D loop contributes to the binding affinity for pembrolizumab. This loop intrudes into the groove formed by the CDR loops of pembrolizumab (Fig. 3a,c). The electron density of the C′D loop in the complex structure of PD-1/nivolumab is also clearly shown, probably because of the crystal packing interaction despite the lack of interaction with nivolumab (Fig. 3b). The structural comparisons of the Fv regions of pembrolizumab and nivolumab before and after binding to PD-1 show little deviation in the conformation of the CDRs and minor adjustments in the side chains involved in the interaction with PD-1, implying that these antibody drugs maintain the CDR loops in productive binding conformations prior to interaction with PD-1 (Supplementary Fig. 1a,b). The high avidity of these two antibodies, which results from the bivalency of IgG, could also contribute to their tight binding to PD-1. Taken together, these structural features suggest that the mechanism by which the anti-PD-1 antibodies block the PD-1/PD-L1 interaction is through outcompeting PD-L1 for binding to PD-1.

### Crystal structure of PD-L1 in complex with BMS-936559 Fab

**Figure 4:**
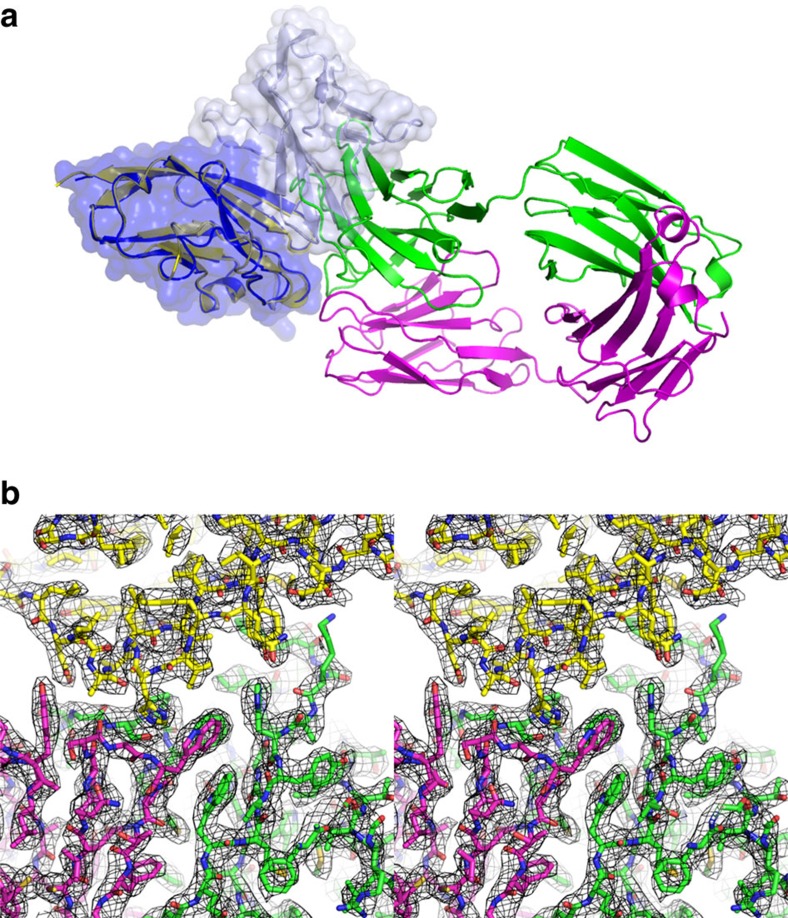
Crystal structure of PD-L1 in complex with BMS-936559. (**a**) Ribbon representation of PD-L1 (yellow) in complex with BMS-936559 Fab fragment (heavy chain: green, light chain: purple). PD-1/PD-L1 complex (PDB code 4zqk) was superimposed onto the PD-L1 molecule in the complex of PD-L1/BMS-936559 with mixed ribbon/surface representation. PD-1 and PD-L1 in PD-1/PD-L1 complex are coloured pale blue and blue, respectively. The orientation of the PD-1/PD-L1 complex is same as that of Fig. 1a,c. (**b**) Stereoview of the 2fo-fc composite omit map (1.2 σ contour level) at the interface between PD-L1 (yellow) and BMS-936559 (heavy chain: green, light chain: purple) calculated at 2.8 Å resolution.

**Figure 5:**
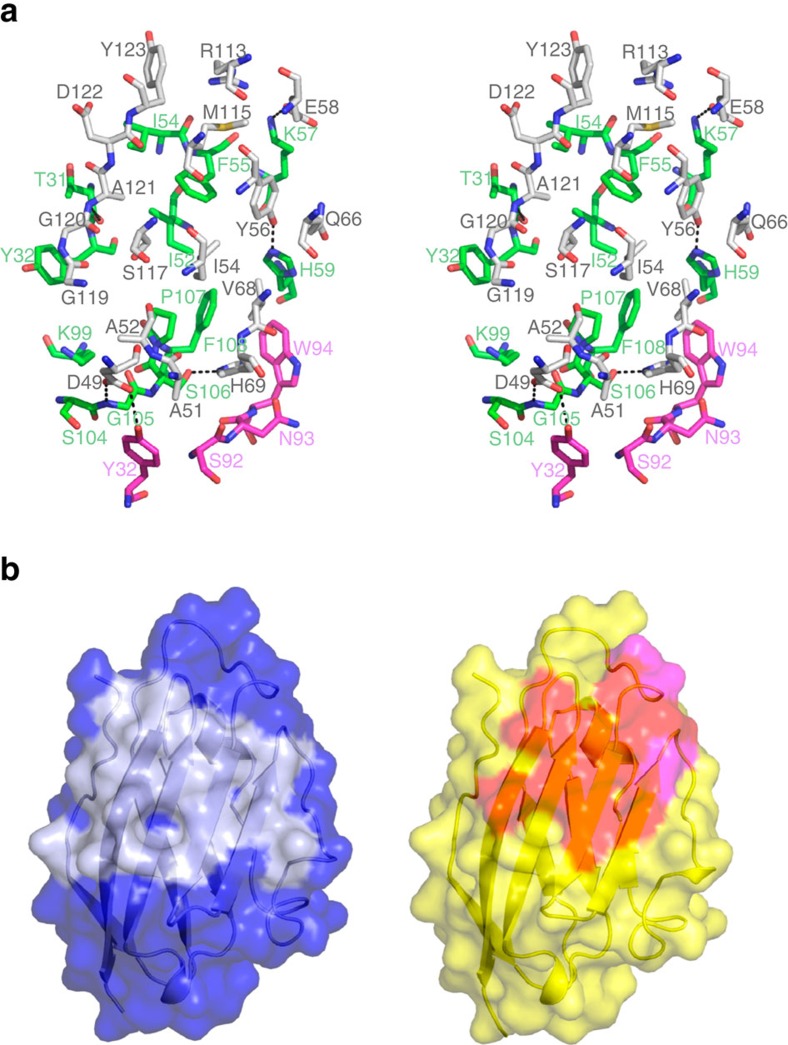
Interaction between PD-L1 and BMS-936559. (**a**) Stereoview of the detailed PD-L1/BMS-936559 interface. The carbon atoms of PD-L1 and heavy and light chain of BMS-936559 are coloured grey, green and purple, respectively. Hydrogen bonds and a salt bridge are indicated with dashed lines. (**b**) Surface representations of the PD-L1 molecules in the complex structures of PD-1/PD-L1 (left) and PD-1/BMS-936559 (right). The surface of PD-L1 in PD-1/PD-L1 is coloured blue and the PD-1 binding site on the surface of PD-L1 is coloured pale blue. The surface of PD-1 in PD-1/BMS-936559 is colored yellow. The epitope regions for the heavy and light chain of BMS-936559 are coloured red and purple, respectively.

BMS-936559, a fully human IgG4 antibody that blocks PD-L1, has been shown to induce objective responses in melanoma, NSCLC, and certain other solid tumours in Phase I clinical trials^34^,^35^. The PD-L1/BMS-936559 Fab fragment complex was crystallized and its structure was determined and refined to a resolution of 2.8 Å (Fig. 4). Superposition of the PD-L1 molecules extracted from PD-1/PD-L1 and PD-L1/BMS-936559 yielded a root mean s.d. of 0.71 Å for all of the Cα atoms, indicating that no significant overall structural difference occurred (Supplementary Fig. 2). The side-chain atoms of _PDL1_D49, _PDL1_Y56 and _PDL1_H69 and main-chain atom of _PDL1_A121 participate in the formation of hydrogen bonds between PD-L1 and BMS-936559, while a salt bridge is formed by _PDL1_E58 (Fig. 5a). Numerous residues of PD-L1, including _PDL1_A51, _PDL1_A53, _PDL1_I54, _PDL1_Y56, _PDL1_Q66, _PDL1_V68, _PDL1_H69, _PDL1_R113, _PDL1_M115, _PDL1_S117, _PDL1_G119, _PDL1_G120, _PDL1_D122 and _PDL1_Y123, are involved in van der Waals contact with BMS-936559. The paratope of BMS-936559 consists of _heavy_T31 and _heavy_Y32 of HCDR1; _heavy_I52, _heavy_I54, _heavy_F55, _heavy_K57, and _heavy_H59 of HCDR2; _heavy_K99, _heavy_S104, _heavy_G105, _heavy_S106, _heavy_P107 and _heavy_F108 of HCDR3; _light_T32 of LCDR1; and _light_S92, _light_N93, and _light_W94 of LCDR3. The total buried surface area of PD-L1/BMS-936559 is 1,349 Å^2^, which is much smaller than that of PD-1/PD-L1 (1,970 Å^2^) (ref. 30). However, the epitope of BMS-936559 occupies a large part of the PD-1 binding site (Fig. 5b); in addition, high avidity, resulting from IgG bivalency, is expected due to the high expression level of PD-L1 in many cancer types^36^, leading to efficient blockade of the PD-1/PD-L1 interaction.

### Crystal structure of CTLA-4 in complex with tremelimumab Fab

**Figure 6:**
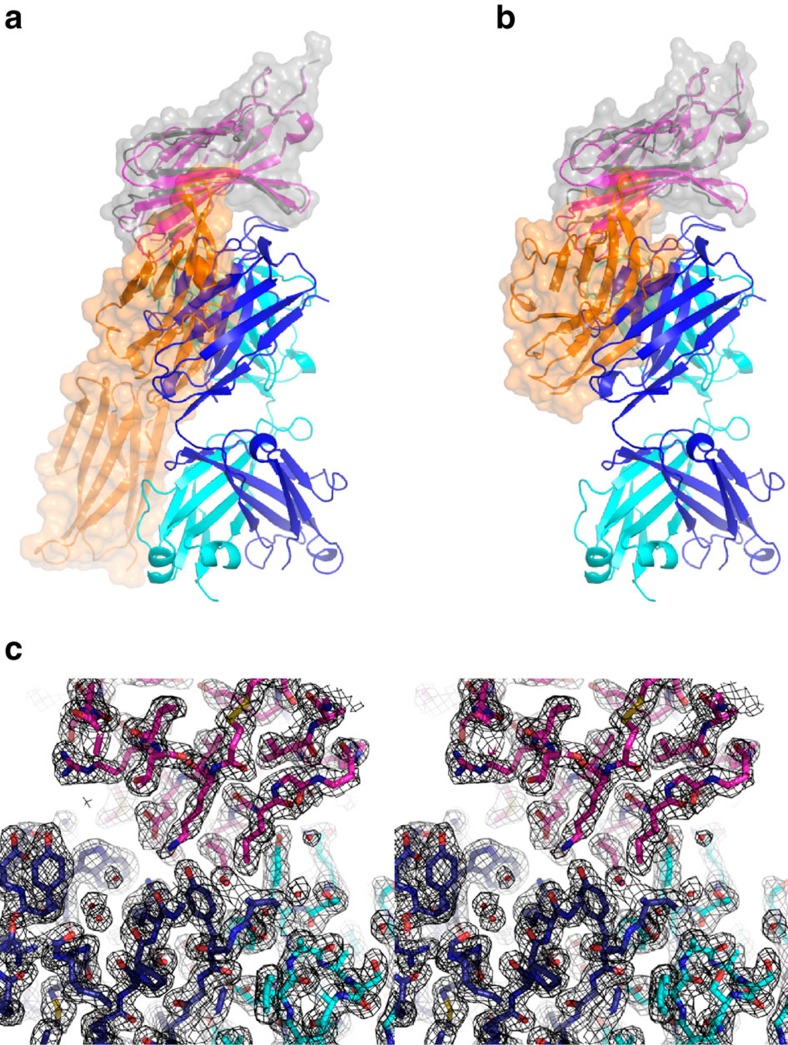
Crystal structure of CTLA-4 in complex with tremelimumab. (**a**,**b**) Ribbon representation of CTLA-4 (purple) in complex with tremelimumab Fab fragment (heavy chain: blue, light chain: cyan). In **a**, the CTLA-4(grey)/B7-1(orange) complex (PDB code 1i8l) was superimposed onto the CTLA-4 molecule in the CTLA-4/tremelimumab complex with a mixed ribbon/surface representation. In **b**, the CTLA-4(grey)/B7-2(orange) complex (PDB code 1i85) was superimposed onto CTLA-4 in the CTLA-4/tremelimumab complex with a mixed ribbon/surface representation. (**c**) Stereoview of the 2fo-fc composite omit map (1.2 σ contour level) at the interface between CTLA-4 (purple) and tremelimumab (heavy chain: blue, light chain: cyan) calculated at 2.0 Å resolution.

**Figure 7:**
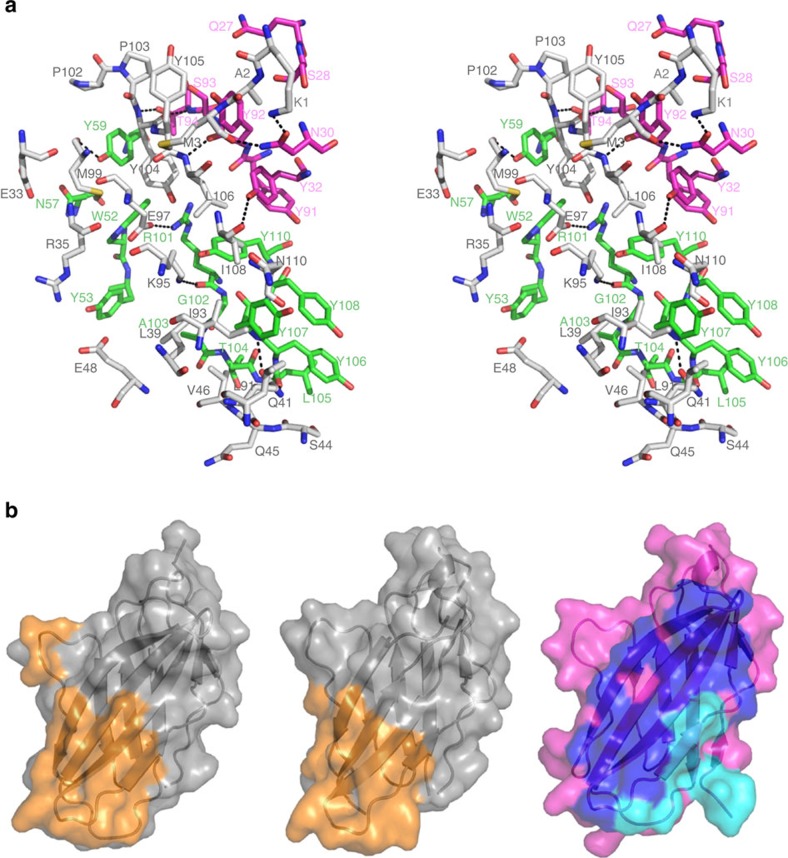
Interaction between CTLA-4 and tremelimumab. (**a**) Stereoview of the detailed CTLA-4/tremelimumab interface. The carbon atoms CTLA-4 and heavy and light chain of tremelimumab are coloured grey, green and purple, respectively. Hydrogen bonds and a salt bridge are indicated with dashed lines. (**b**) Surface representations of the CTLA-4 molecules in the complex structures of CTLA-4/B7-1 (left), CTLA-4/B7-2 (centre) and CTLA-4/tremelimumab (right). The surfaces of CTLA-4 in CTLA-4/B7-1 and CTLA-4/B7-2 are coloured grey and the B7-1 or B7-2 binding site on the surface of CTLA-4 is coloured orange. The surface of CTLA-4 in CTLA-4/tremelimumab is colored purple. The epitope regions for the heavy and light chain of tremelimumab are coloured blue and cyan, respectively.

Tremelimumab is a fully human IgG2 antibody that blocks CTLA-4. In 2015, tremelimumab was granted Fast Track Designation and Orphan Drug Designation by the FDA as a potential treatment for malignant mesothelioma. In addition, tremelimumab is being studied in combination with an anti-PD-L1, durvalumab, in multiple tumour types^37^. The CTLA-4/tremelimumab Fab complex and free tremelimumab Fab were crystallized and their structures were determined and refined to a resolution of 2.0 Å and 2.3 Å, respectively (Fig. 6). The tremelimumab epitope of CTLA-4 consists of several discontinuous segments (Fig. 7a). The side chain atoms of _CTLA4_K1 and _CTLA4_K95 and main chain atoms of _CTLA4_M3, _CTLA4_Q41, _CTLA4_M99, _CTLA4_Y104, _CTLA4_L106, and _CTLA4_I108 participate in the formation of hydrogen bonds between CTLA-4 and tremelimumab, whereas _CTLA4_E97 participates in salt bridge formation. A large number of residues, including _CTLA4_A2, _CTLA4_E33, _CTLA4_R35, _CTLA4_S44, _CTLA4_Q45, _CTLA4_V46, _CTLA4_E48, _CTLA4_L91, _CTLA4_I93, _CTLA4_M99, _CTLA4_P102, _CTLA4_P103, _CTLA4_Y104, _CTLA4_Y105, _CTLA4_L106, _CTLA4_I108, and _CTLA4_N110, contribute to van der Waals contact with tremelimumab. The tremelimumab residues involved in the interaction with CTLA-4 are _heavy_W52, _heavy_Y53, _heavy_N57 and _heavy_Y59 of HCDR2; _heavy_R101, _heavy_G102, _heavy_A103, _heavy_T104, _heavy_L105, _heavy_Y106, _heavy_Y107, _heavy_Y108 and _heavy_Y110 of HCDR3; _light_Q27, _light_S28, _light_N30 and _light_Y32 of LCDR1; and _light_Y91, _light_Y92, _light_S93 and _light_T94 of LCDR3. Superposition of the CTLA-4 molecules extracted from CTLA-4/tremelimumab and CTLA-4/B7-1 yielded a root mean square deviation of 1.08 Å for all of the Cα atoms, indicating no significant deviation in the two structures except for several residues at the antibody–antigen interface, which are not involved in the interaction with B7 ligands (Supplementary Fig. 3). As observed in the structure of free CTLA-4 and that of CTLA-4 in complex with B7-1/2, the three consecutive proline residues of the MYPPPYY (residues 99–105) sequence of the FG loop of CTLA-4 in complex with tremelimumab adopt an unusual *cis-trans-cis* conformation and participate in interactions with the antibody^28^,^29^,^38^ (Fig. 7a). The tremelimumab epitope partially occupies the B7-1/2 binding site of CTLA-4, with a larger interface area between CTLA-4 and tremelimumab (buried surface area of 1,802 Å^2^) than the receptor–ligand interface (1,255 Å^2^ for CTLA-4/B7-1 and 1,212 Å^2^ for CTLA-4/B7-2) (Fig. 7b). Therefore, the binding of tremelimumab to CTLA-4 efficiently competes with B7-1/2 binding to CTLA-4, thereby blocking the function of CTLA-4 in cancer.

## Discussion

Checkpoint-blocking antibodies have ensured significant advances in cancer therapy and provided a new weapon against cancer. The durability of this immunotherapy largely exceeds that of other forms of anticancer therapy and sometimes enables long-term remission where patients exhibit no clinical signs of cancer for many years. However, monotherapies of these drugs elicit the therapeutic benefit only in a fraction of patients treated. As a critical next step in the progress toward improving clinical responses, combination immunotherapies have been intensively investigated^39^,^40^,^41^. Combinations of anti-CTLA-4 or anti-PD-1/PD-L1 therapies with other immunotherapies, chemotherapies, targeted therapies, hormonal therapies and radiation therapies are being explored. The combination of anti-CTLA-4 and anti-PD-1 has been the most successful of these combinations tested so far, probably because CTLA-4 and PD-1 modulate the activity of T-cells at different stages of T-cell immunity^16^,^26^. Thus, dual blockade of CTLA-4 and PD-1 can be a reasonable and synergistic combination therapy based on their distinct mechanisms of action. In addition, structural studies of therapeutic antibodies to reveal their precise epitopes and therapeutic mechanisms can also facilitate a rational design of combination immunotherapies, as different epitopes and molecular mechanisms of therapeutic antibodies can lead to different therapeutic effects even when therapeutic antibodies target the same molecule^42^. Pembrolizumab and nivolumab partially share epitopes and three-dimensional (3D) space when binding to PD-1, implying that the mechanisms of antagonism of the two antibodies are highly similar (Supplementary Fig. 4). These results are consistent with earlier clinical data showing that the response rate for both drugs appears similar^26^; thus, little synergistic therapeutic effect should be expected from a combination of only these two drugs.

**Figure 8:**
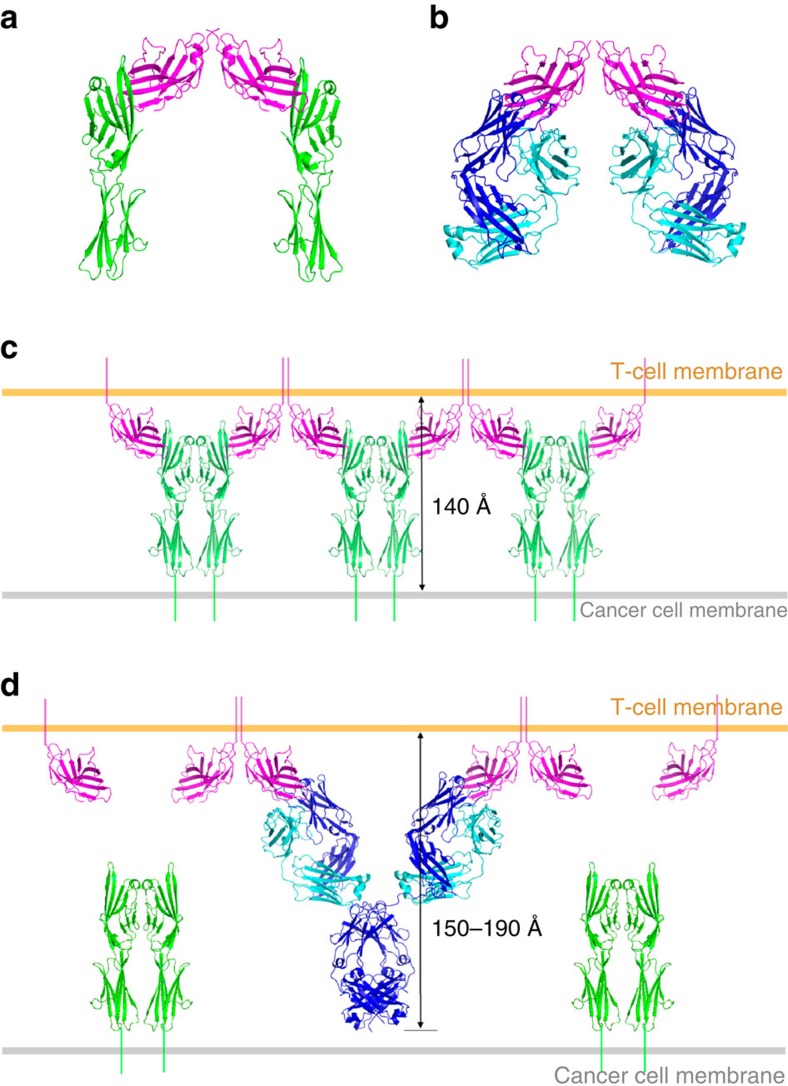
Hypothetical model for prevention of an alternating arrangement of bivalent dimers of CTLA-4 and B7-1/2 by tremelimumab binding. (**a**) Structure of CTLA-4 dimer (purple) binding to two B7-1 molecules (green) (PDB code 1i8l). (**b**) Structure of CTLA-4 dimer (purple) binding to two tremelimumab Fab fragments (heavy chain: blue, light chain: cyan) in the crystal. (**c**) Suggested model for an alternating periodic arrangement of bivalent dimers of CTLA-4 and B7-1 at the interface between a T-cell and cancer cell, imposing an intercellular distance of ∼140 Å^23^,^24^. (**d**) Suggested model for the bivalent interaction of tremelimumab with CTLA-4. Tremelimumab binds two CTLA-4 molecules in the vicinity of the two binding site of IgG, spanning 150–190 Å in the perpendicular direction from the T-cell membrane.

CTLA-4 exists as a constitutive homodimer generated by an intermolecular disulphide bond^43^ (Fig. 8a). Although a single CTLA-4/tremelimumab Fab complex exists in an asymmetric unit, a crystallographic two-fold symmetry generates a homodimeric interface of CTLA-4, which is essentially identical to that observed in the structures of free CTLA-4 or CTLA-4 in complex with B7-1, B7-2, and engineered lipocalin 2 (refs 28, 29, 38, 44), implying that tremelimumab binding does not affect the homodimerization of CTLA-4 (Fig. 8b). In the crystal structures of CTLA-4 in complex with B7-1 or B7-2, the crystal packing generates an alternating periodic arrangement in which bivalent CTLA-4 homodimers connect bivalent B7-1/2 homodimers, providing a model describing the assembly of CTLA-4 and B7-1/2 at the interface between a T-cell and cancer cell^28^,^29^ (Fig. 8c). In this model, the CTLA-4/B7 complex is expected to span a distance of ∼140 Å, which includes the stalk regions connecting these molecules to the opposing cell membranes. This distance is compatible with other ligand–receptor interactions at the central zone of the immunological synapse, suggesting that this oligomeric arrangement of CTLA-4/B7 complex can facilitate the efficient use of low-abundance CTLA-4 at the T-cell surface, thereby promoting the inhibitory signalling of CTLA-4 and decreasing the local concentration of CD28 through simple steric crowding^28^,^29^,^45^. As tremelimumab is an IgG2 antibody, it is capable of binding two CTLA-4 molecules with high avidity through Y-shaped bivalent presentation; the dimension of the CTLA-4/tremelimumab complex is estimated to span 150–190 Å. This distance is incompatible with the oligomeric array of the CTLA-4/B7 complex (Fig. 8d). Therefore, in addition to simple antagonism of the interaction between CTLA-4 and B7-1/2, tremelimumab binding could prevent or disrupt the uniquely organized assembly of the CTLA-4/B7 complex within the immunological synapse.

**Figure 9:**
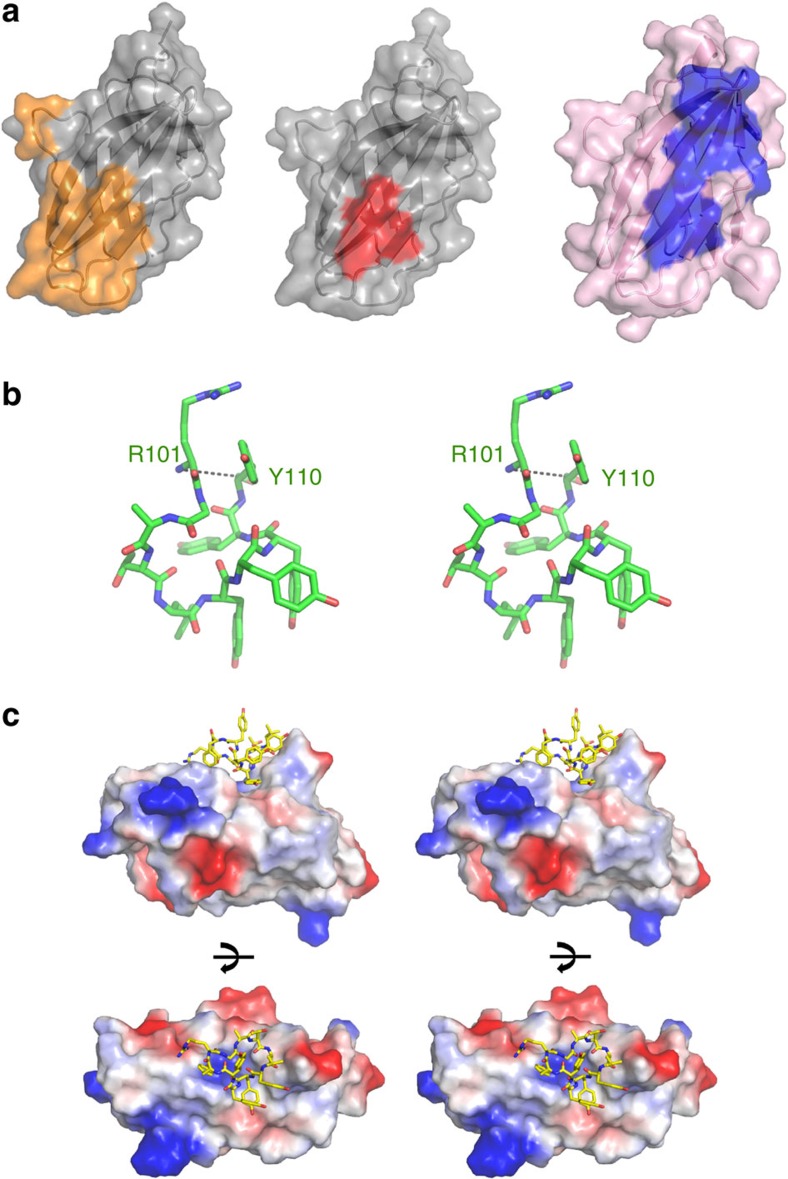
Suggestion of a cyclic peptide as a modulator of the CTLA-4/B7 interaction. (**a**) Surface representations of the CTLA-4 molecule in the CTLA-4/B7-1 complex (left, PDB code 1i8l). The B7-1 binding site on the surface of CTLA-4 is coloured orange. Surface representations of CTLA-4 in the complex structures of CTLA-4/tremelimumab (right). The HCDR3 loop binding site on the surface of CTLA-4 is coloured blue. The overlapping region between the binding sites of B7-1 and HCDR3 is coloured red on the surface of CTLA-4 (centre). (**b**) Stereoview of the HCDR3 loop in the structure of CTLA-4/tremelimumab. The dashed line suggests a peptide bond between R101 and Y110 for generating a cyclic peptide. (**c**) Stereoview of a hypothetical model of the interaction between CTLA-4 (electrostatic surface representation) and the cyclic peptide based on the HCDR3 sequence of tremelimumab (yellow stick). The cyclic peptide was generated by connecting R101 and Y110 of the HCDR3 loop in the structure of CTLA-4/tremelimumab through a peptide bond, and its structure was polished by regularization and real space refinement in COOT (ref. 52). In this model, the cyclic peptide retains all of the interactions of HCDR3 in the structure of CTLA-4/tremelimumab.

Despite the clinical success of checkpoint-blocking antibodies, biologics such as therapeutic antibodies have several shortcomings, including the high cost, limited half-life and immunogenicity caused by multiple dosages. As an alternative treatment strategy to overcome the drawbacks of passive antibody-based therapy, several small-molecule immunomodulators targeting the PD-1/PD-L1 axis have been reported^46^,^47^,^48^,^49^,^50^. While most of them are based on peptidomimetics, a recent structural study with the first nonpeptidic chemical inhibitor of the PD-1/PD-L1 interaction revealed that this compound binds to PD-L1 and induces dimerization of PD-L1, thus occluding the PD-1 binding site of PD-L1 (ref. 51). Interestingly, the complex structure of CTLA-4/tremelimumab Fab provided insight for the design of a small-molecule modulator that interferes with the CTLA-4/B7 signal pathway. Nine of ten residues in the HCDR3 loop (residues 101–110 of the heavy chain) of tremelimumab are involved in the interaction with CTLA-4 (Fig. 7a). In addition, the binding site of the HCDR3 loop overlaps part of the binding site of B7-1/2 on the surface of CTLA-4 (Fig. 9a). Additionally, _heavy_R101 and _heavy_Y110 are located closely enough to be connected directly by a peptide bond (Fig. 9b). This extensive interaction and the structural features of the HCDR3 loop in the CTLA-4/tremelimumab complex suggest that cyclic peptides based on the HCDR3 sequence may also prevent binding of B7-1/2 to CTLA-4 (Fig. 9c). As the HCDR3 loop conformation in the complex is essentially identical to that of tremelimumab Fab alone, other CDRs and framework regions of tremelimumab likely contribute to the conformation of HCDR3, implying that antibody context is important for the proper display of the tremelimumab HCDR3 (Supplementary Fig. 1c). If this is indeed the case, a cyclic peptide approach would not be so promising because of its intrinsic conformational flexibility. Nonetheless, a cyclic peptide or peptidomimetic approach based on the HCDR3 sequence in combination with the power of phage-display library technology may facilitate the discovery of small-molecule modulators of the CTLA-4/B7 interaction, providing an alternative treatment strategy to overcome the drawbacks of antibody–based drugs.

In summary, the elucidation of the crystal structures of the checkpoint molecules PD-1, PD-L1 and CTLA-4 in complex with the Fab fragments of therapeutic antibodies sheds light on the molecular mechanisms underlying the therapeutic activity of these monoclonal antibodies. In addition, the precise epitopes revealed by the present complex structures should provide useful information for the improvement of current therapeutic antibodies and development of more effective immunotherapeutic combination strategies and small-molecule modulators that can attenuate checkpoint signalling.

## Methods

### Expression and purification of checkpoint proteins

Genes encoding the ectodomains of human PD-1 (aa 26–150), human PD-L1 (aa 18–134) and human CTLA-4 (aa 1–126) were subcloned into pET-21a (Novagen), respectively. A Cys to Ser mutation was introduced at position 93 of PD-1 to aid expression and folding. All proteins were expressed in *E. coli* BL21(DE3) as inclusion bodies. The cells were grown at 37 °C in LB medium supplemented with 50 μg ml^−1^ ampicillin until OD_600_ reached 0.6–1.0, and the protein expression was induced with 1 mM IPTG and incubated for 4 h at 37 °C. The cells were harvested by centrifugation, re-suspended in lysis buffer (20 mM Tris, pH 8.0, 200 mM NaCl) and lysed by sonication on ice. Inclusion bodies were recovered by centrifugation (25,000*g* for 0.5 h at 4 °C) and solubilized in 8 M urea, 20 mM Tris, pH 8.0, 200 mM NaCl by stirring overnight. After removing undissolved residue by centrifugation (25,000*g* for 0.5 h at 4 °C), solubilized fraction was applied to HisTrap HP column (GE Healthcare Life Sciences) and washed with five column volumes of wash buffer (8 M urea, 20 mM Tris, pH 8.0, 200 mM NaCl, 50 mM imidazole). The protein was then eluted with elution buffer (8 M urea, 20 mM Tris, pH 8.0, 200 mM NaCl, 400 mM imidazole). The eluted protein was refolded by dialysis three times against 20 mM Tris, pH 8.0, 200 mM NaCl and purified further by gel filtration chromatography using a HiLoad 16/60 Superdex 200 pg column (GE Healthcare Life Sciences). The protein purity was evaluated by reducing and nonreducing SDS–PAGE.

### Expression and purification of Fab fragments

The DNA sequences for the Fab fragments of pembrolizumab, nivolumab, BMS-936559 and tremelimumab were synthesized after codon-optimization for expression in *E. coli* (Bioneer, Inc). The sequences for heavy chain and light chain were cloned into a modified pBAD vector, containing the STII signal sequence in each chain for periplasmic secretion and a C-terminal 6His-tag in heavy chain. The plasmid pBAD-Fab was transformed into *E. coli* Top10F (Invitrogen). The cells were grown at 37 °C in LB medium supplemented with 50 μg ml^−1^ ampicillin. At an OD_600_ of 1.0, the protein expression was induced with 0.2% arabinose and cells were grown at 30 °C for 15 h. The cells were harvested by centrifugation, re-suspended in lysis buffer (20 mM Tris, pH 8.0, 200 mM NaCl) and lysed by sonication on ice. After removing cell debris by centrifugation (25,000*g* for 0.5 h at 4 °C), the supernatant containing soluble protein was applied to HisTrap HP column (GE Healthcare Life Sciences) and washed with five column volumes of wash buffer (20 mM Tris, pH 8.0, 300 mM NaCl, 50 mM imidazole). The protein was then eluted with elution buffer (20 mM Tris, pH 8.0, 300 mM NaCl, 400 mM imidazole). The eluted protein was concentrated for gel filtration chromatography using a HiLoad 16/60 Superdex 200 pg column (GE Healthcare Life Sciences). The column had previously been equilibrated with gel filtration buffer (20 mM Tris, pH 8.0, 300 mM NaCl). The elution profile of the protein showed a single major peak and the protein quality was evaluated by reducing and nonreducing SDS–PAGE.

### Crystallization and structure determination

Details of the crystallization and structure determination of the nivolumab Fab, tremelimumab Fab, PD-1/pembrolizumab Fab complex, PD-1/nivolumab Fab complex, PD-L1/BMS-936559 Fab complex, CTLA-4/tremelimumab Fab complex are described in Supplementary Methods. Data collection and refinement statistics are summarized in Table 1.

### Data availability

The atomic coordinates and structure factors have been deposited in the Protein Data Bank under accession codes 5GGQ, 5GGR, 5GGS, 5GGT, 5GGU and 5GGV. All additional experimental data are available from the corresponding author on request.

## Additional information

**How to cite this article:** Lee, J. Y. *et al*. Structural basis of checkpoint blockade by monoclonal antibodies in cancer immunotherapy. *Nat. Commun.* **7,** 13354 doi: 10.1038/ncomms13354 (2016).

**Publisher's note:** Springer Nature remains neutral with regard to jurisdictional claims in published maps and institutional affiliations.

## Supplementary Material

Supplementary Information Supplementary Figures 1-4, Supplementary Methods and Supplementary References.

## Figures and Tables

**Table 1:**
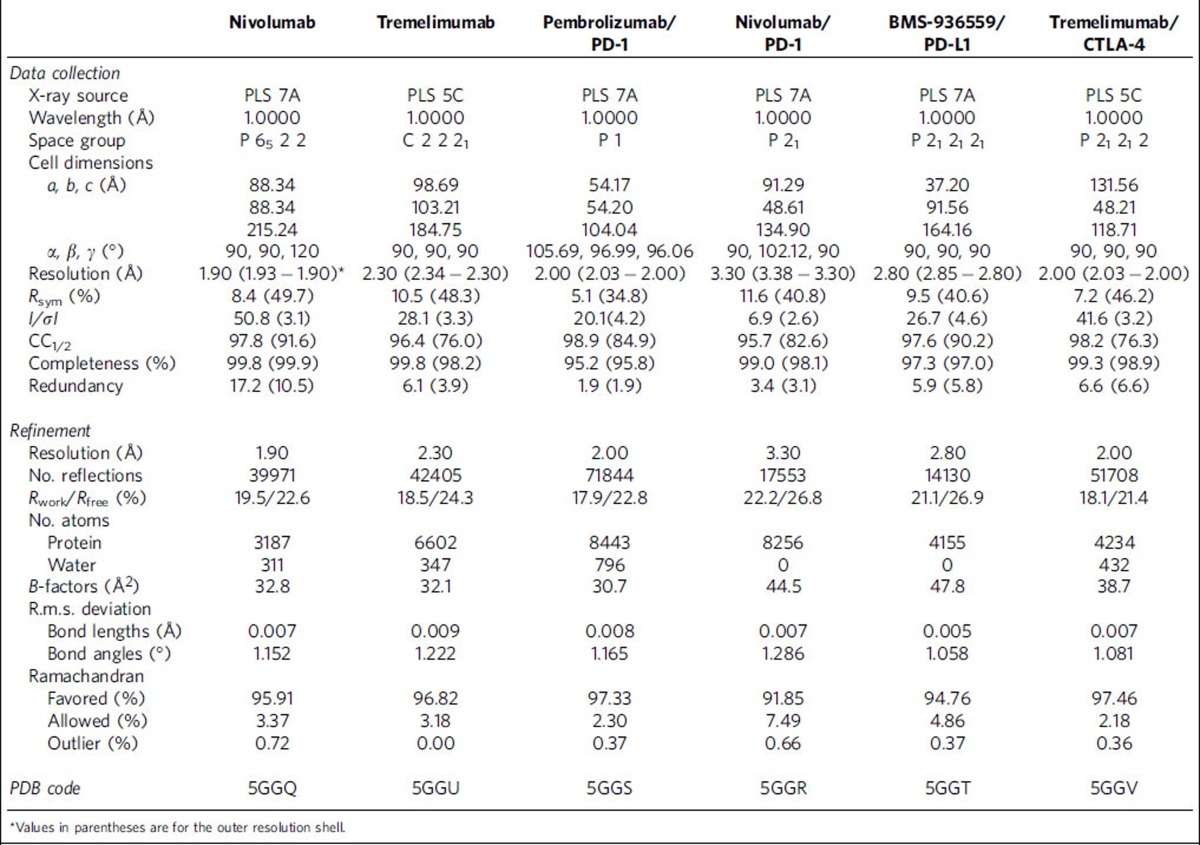
Data collection and refinement statistics.
