# Supplementary material for: Structural basis of checkpoint blockade by monoclonal antibodies in cancer immunotherapy
Source: Nat Commun. 2016 Oct 31;7:13354. doi: 10.1038/ncomms13354 (PMC5095608; doi:10.1038/ncomms13354)
Supplement: Supplementary Information — Supplementary Figures 1-4, Supplementary Methods and Supplementary References. [file ncomms13354-s1.pdf]

SUPPLEMENTARY FIGURES

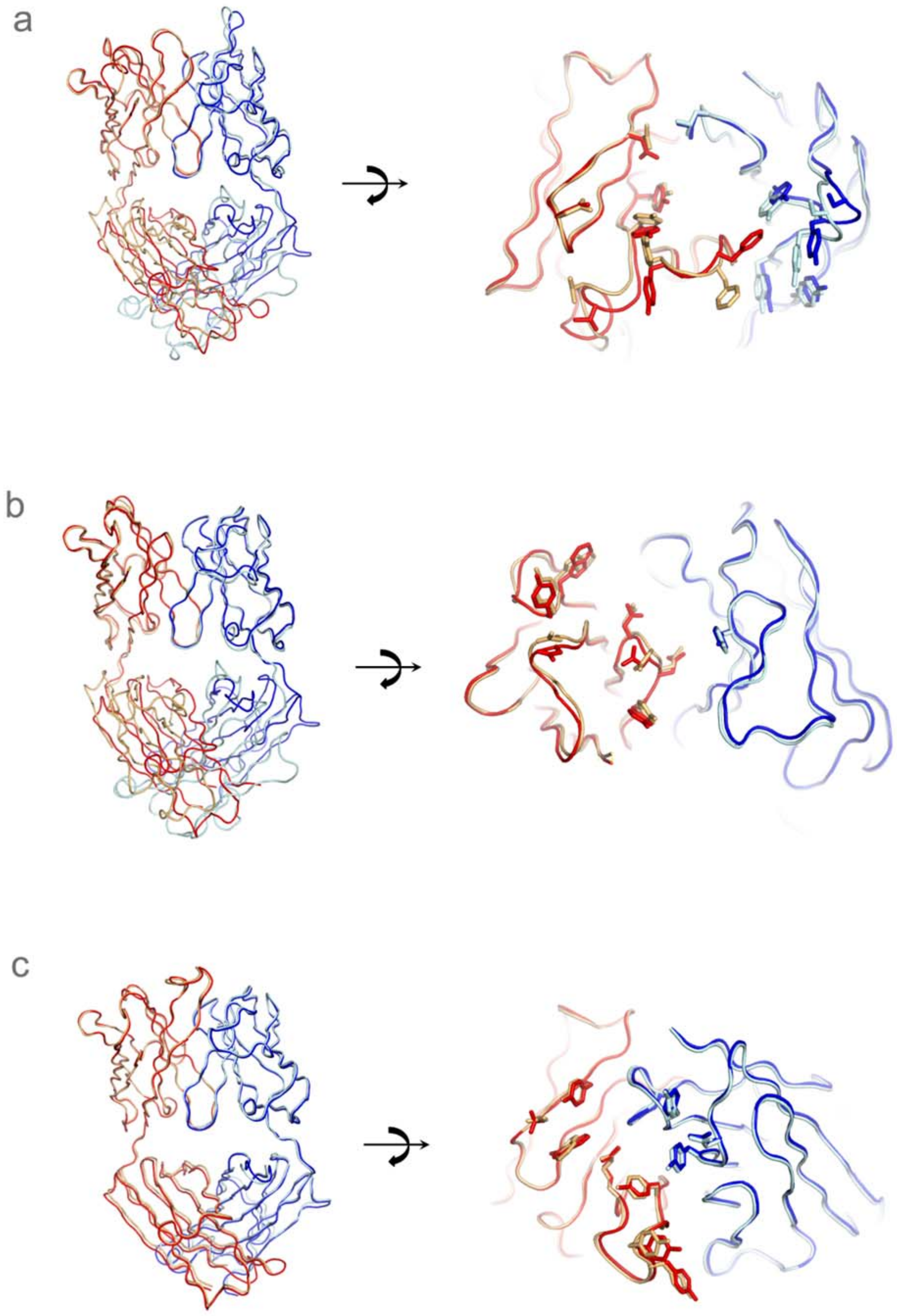

Supplementary Figure 1. Structural comparison of the Fv regions before and after binding to checkpoint molecules.

a. Superposition of the Fv region of free pembrolizumab Fab (PDB code 5dk3) onto that of pembrolizumab in complex with PD-1.

b. Superposition of the Fv region of free nivolumab Fab onto that of nivolumab in complex with PD-1.

c. Superposition of the Fv region of free tremelimumab Fab onto that of tremelimumab in complex with CTLA-4.

In **a**, **b**, and **c**, the heavy and light chains of the Fab fragments in the complexes are colored red and blue, respectively. The heavy and light chains in free form are colored yellow and pale blue, respectively.

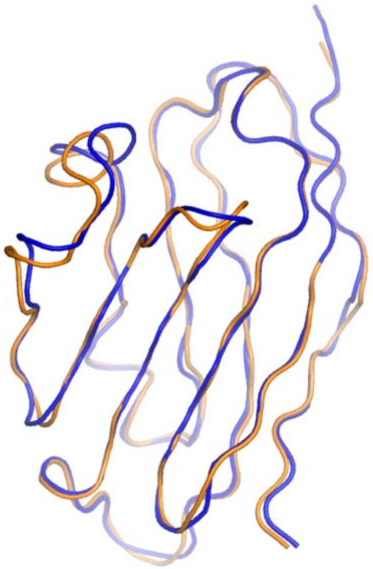

Supplementary Figure 2. Superposition of the PD-L1 molecules extracted from PD-1/PD-L1 (orange, PDB code 4zqk) and PD-L1/BMS-936559 (blue). The comparison shows little deviation in the two structures, yielding r.m.s. deviation of 0.71 Å.

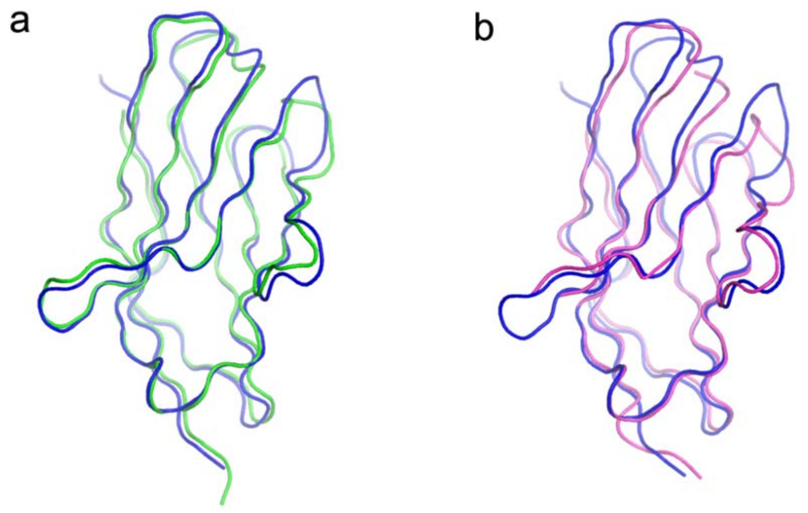

Supplementary Figure 3. Structural comparison of the CTLA-4 molecules extracted from CTLA-4/tremelimumab and CTLA-4/B7 ligands.

a. Superposition of the CTLA-4 molecules extracted from CTLA-4/tremelimumab (blue) and CTLA-4/B7-1 (green, PDB code 1i8l), yielding r.m.s. deviation of 1.08 Å.

b. Superposition of the CTLA-4 molecules extracted from CTLA-4/tremelimumab (blue) and CTLA-4/B7-2 (red, PDB code 1i85), yielding r.m.s. deviation of 1.54 Å.

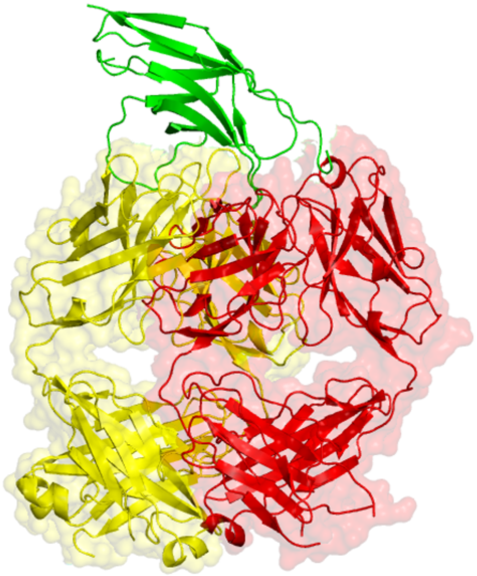

Supplementary Figure 4. Comparison of binding of pembrolizumab and nivolumab to PD-1.

The 3D spaces occupied by pembrolizumab (yellow) and nivolumab (red) partially overlap when binding to PD-1 (green); therefore, these two antibody drugs cannot bind to a PD-1 molecule at the same time.

SUPPLEMENTARY METHODS

Crystallization and structure determination of the nivolumab Fab

Gel-filtration fractions containing the nivolumab Fab was concentrated to 6 mg ml^{-1} in 20 mM Tris, pH 8.0, and 300 mM NaCl. Crystals were grown using hanging-drop vapor diffusion with a reservoir solution containing 0.1 M citric acid, pH 3.5, 2.0 M ammonium sulfate at 20 °C within 7 days. Crystals were cryoprotected by brief immersion in well solution supplemented with 25% ethylene glycol, and flash frozen in liquid nitrogen. X-ray diffraction data were collected at 100 K on beamline 7A of the Pohang Light Source (PLS), Republic of Korea. The crystals belonged to space group $P6_522$ (a = b = 88.34, c = 215.24 Å) with one copy in the asymmetric unit. X-ray diffraction data were collected to a resolution of 1.90 Å, integrated and scaled using HKL2000 (HKL Research) [1]. The structure was solved by molecular replacement using Phaser[2] with a structure of Fab that has high sequence identities with nivolumab Fab (PDB code 3eyq, chains C and D). Due to the intrinsic elbow flexibility of a Fab fragment, the Fv region and the other region including C_{H1} and C_L domain were separated when used as a search model. At this point, the electron density corresponding nivolumab was prominent. Iterative rounds of refinement were done using PHENIX[3] with manual inspection using COOT[4]. Statistics for data collection and refinement can be found in Table 1. The final model was deposited in the Protein Data Bank under accession number 5GGQ.

Crystallization and structure determination of the tremelimumab Fab

Gel-filtration fractions containing the tremelimumab Fab was concentrated to 8 mg ml^{-1} in

20 mM Tris, pH 8.0, and 300 mM NaCl. Crystals were grown using hanging-drop vapor diffusion with a reservoir solution containing 50 mM Tris, pH 8.5, 30% PEG4,000, 0.2 M ammonium chloride, 10 mM $CaCl_2$ at 20 °C within 7 days. Crystals were cryoprotected by brief immersion in well solution supplemented with 15% glycerol, and flash frozen in liquid nitrogen. X-ray diffraction data were collected at 100 K on beamline 5C of the Pohang Light Source (PLS), Republic of Korea. The crystals belonged to space group $C222_1$ (a = 98.69, b = 103.21, c = 184.75 Å) with two copies in the asymmetric unit. X-ray diffraction data were collected to a resolution of 2.30 Å, integrated and scaled using HKL2000 (HKL Research). The structure was solved by molecular replacement using Phaser with a structure of Fab that has high sequence identities with tremelimumab Fab (PDB code 4xwg, chains H and L). Due to the intrinsic elbow flexibility of a Fab fragment, the Fv region and the other region including C_{H1} and C_L domain were separated when used as a search model. At this point, the electron density corresponding tremelimumab was prominent. Iterative rounds of refinement were done using PHENIX with manual inspection using COOT. Statistics for data collection and refinement can be found in Table 1. The final model was deposited in the Protein Data Bank under accession number 5GGU.

Crystallization and structure determination of the PD-1/pembrolizumab Fab complex

Purified PD-1 and pembrolizumab Fab were mixed in 1:1 molar ratio and incubated for 1 h at 4 °C before being subjected to size exclusion chromatography using a HiLoad 16/60 Superdex 200 pg column (GE Healthcare) equilibrated with 20 mM Tris, pH 8.0, and 300 mM NaCl. Gel-filtration fractions containing the PD-1/pembrolizumab Fab complex was concentrated to 7 mg ml^{-1} in 20 mM Tris, pH 8.0, and 300 mM NaCl. Crystals were grown using hanging-drop vapor diffusion with a reservoir solution containing 100 mM MES, pH

6.5, 12% PEG500 MME, 6% PEG20,000, 50 mM ammonium acetate at 20 °C within 20 days. Crystals were cryoprotected by brief immersion in well solution supplemented with 10% glycerol, and flash frozen in liquid nitrogen. X-ray diffraction data were collected at 100 K on beamline 7A of the Pohang Light Source (PLS), Republic of Korea. The crystals belonged to space group P1 (a = 54.17, b = 54.20, c = 104.04 Å, α = 105.69, β = 96.99, γ = 96.06 ˚) with two copies in the asymmetric unit. X-ray diffraction data were collected to a resolution of 2.00 Å, integrated and scaled using HKL2000 (HKL Research). The structure was solved by molecular replacement using Phaser with a structure of Fab that has high sequence identities with pembrolizumab Fab (PDB code 3c08, chains H and L) and human PD-1 (PDB code 3rrq). Due to the intrinsic elbow flexibility of a Fab fragment, the Fv region and the other region including C_{H1} and C_L domain were separated when used as a search model. At this point, the electron density corresponding the PD-1/pembrolizumab Fab complex was prominent. Iterative rounds of refinement were done using PHENIX with manual inspection using COOT. Statistics for data collection and refinement can be found in Table 1. The final model was deposited in the Protein Data Bank under accession number 5GGS.

Crystallization and structure determination of the PD-1/nivolumab Fab complex

Purified PD-1 and nivolumab Fab were mixed in 1:1 molar ratio and incubated for 1 h at 4 °C before being subjected to size exclusion chromatography using a HiLoad 16/60 Superdex 200 pg column (GE Healthcare) equilibrated with 20 mM Tris, pH 8.0, and 300 mM NaCl. Gel-filtration fractions containing the PD-1/nivolumab Fab complex was concentrated to 4 mg ml^{-1} in 20 mM Tris, pH 8.0, and 300 mM NaCl. Crystals were grown using hanging-drop vapor diffusion with a reservoir solution containing 100 mM bicine, pH 8.5, 12% PEG500 MME, 6% PEG20,000, 100 mM ammonium acetate at 20 °C within 20 days.

Crystals were cryoprotected by brief immersion in well solution supplemented with 10% glycerol, and flash frozen in liquid nitrogen. X-ray diffraction data were collected at 100 K on beamline 7A of the Pohang Light Source (PLS), Republic of Korea. The crystals belonged to space group $P2_1$ (a = 91.29, b = 48.61, c = 134.90 Å, β = 102.12 ˚) with two copies in the asymmetric unit. X-ray diffraction data were collected to a resolution of 3.30 Å, integrated and scaled using HKL2000 (HKL Research). The structure was solved by molecular replacement using Phaser with a structure of the free nivolumab Fab and human PD-1 (PDB code 3rrq). Due to the intrinsic elbow flexibility of a Fab fragment, the Fv region and the other region including C_{H1} and C_L domain were separated when used as a search model. At this point, the electron density corresponding the PD-1/nivolumab Fab complex was prominent. Iterative rounds of refinement were done using PHENIX with manual inspection using COOT. Statistics for data collection and refinement can be found in Table 1. The final model was deposited in the Protein Data Bank under accession number 5GGR.

Crystallization and structure determination of the PD-L1/BMS-936559 Fab complex

Purified PD-L1 and BMS-936559 Fab were mixed in 1:1 molar ratio and incubated for 1 h at 4 °C before being subjected to size exclusion chromatography using a HiLoad 16/60 Superdex 200 pg column (GE Healthcare) equilibrated with 20 mM Tris, pH 8.0, and 300 mM NaCl. Gel-filtration fractions containing the PD-L1/BMS-936559 Fab complex was concentrated to 6 mg ml^{-1} in 20 mM Tris, pH 8.0, and 300 mM NaCl. Crystals were grown using hanging-drop vapor diffusion with a reservoir solution containing 0.1M imidazole, pH 9.0, 1.2 M sodium citrate at 20 °C within 20 days. Crystals were cryoprotected by brief immersion in well solution supplemented with 25% ethylene glycol, and flash frozen in liquid nitrogen. X-ray diffraction data were collected at 100 K on beamline 7A of the Pohang Light

Source (PLS), Republic of Korea. The crystals belonged to space group $P2_12_12_1$ (a = 37.20, b = 91.56, c = 164.16 Å) with one copy in the asymmetric unit. X-ray diffraction data were collected to a resolution of 2.80 Å, integrated and scaled using HKL2000 (HKL Research). The structure was solved by molecular replacement using Phaser with a structure of Fab that has high sequence identities with BMS-936559 Fab (PDB code 3qot, chains H and L) and human PD-L1 (PDB code 3bik, chain A, residues 18-134). Due to the intrinsic elbow flexibility of a Fab fragment, the Fv region and the other region including C_{H1} and C_L domain were separated when used as a search model. At this point, the electron density corresponding the PD-L1/BMS-936559 Fab complex was prominent. Iterative rounds of refinement were done using PHENIX with manual inspection using COOT. Statistics for data collection and refinement can be found in Table 1. The final model was deposited in the Protein Data Bank under accession number 5GGT.

Crystallization and structure determination of the CTLA-4/tremelimumab Fab complex

Purified CTLA-4 and tremelimumab Fab were mixed in 1:1 molar ratio and incubated for 1 h at 4 °C before being subjected to size exclusion chromatography using a HiLoad 16/60 Superdex 200 pg column (GE Healthcare) equilibrated with 20 mM Tris, pH 8.0, and 300 mM NaCl. Gel-filtration fractions containing the CTLA-4/tremelimumab Fab complex was concentrated to 5 mg ml^{-1} in 20 mM Tris, pH 8.0, and 300 mM NaCl. Crystals were grown using hanging-drop vapor diffusion with a reservoir solution containing 0.1M sodium citrate tribasic dihydrate, pH 5.6, 12% PEG4,000, 100 mM NaCl at 20 °C within 20 days. Crystals were cryoprotected by brief immersion in well solution supplemented with 25% glycerol, and flash frozen in liquid nitrogen. X-ray diffraction data were collected at 100 K on beamline 5C of the Pohang Light Source (PLS), Republic of Korea. The crystals belonged to

space group $P2_12_12$ (a = 131.56, b = 48.21, c = 118.71 Å) with one copy in the asymmetric unit. X-ray diffraction data were collected to a resolution of 2.00 Å, integrated and scaled using HKL2000 (HKL Research). The structure was solved by molecular replacement using Phaser with a structure of the free tremelimumab Fab and human CTLA-4 (PDB code 1i8l, chain C). Due to the intrinsic elbow flexibility of a Fab fragment, the Fv region and the other region including C_{H1} and C_L domain were separated when used as a search model. At this point, the electron density corresponding the CTLA-4/tremelimumab Fab complex was prominent. Iterative rounds of refinement were done using PHENIX with manual inspection using COOT. Statistics for data collection and refinement can be found in Table 1. The final model was deposited in the Protein Data Bank under accession number 5GGV.

SUPPLEMENTARY REFERENCES

1. Otwinowski, Z. & Minor, W. Processing of X-ray diffraction data collected in oscillation mode. *Method Enzymol.* **276**, 307-326 (1997).

2. McCoy, A. J. *et al*. Phaser crystallographic software. *J. Appl. Crystallogr*. **40**, 658–674 (2007)

3. Adams, P. D. *et al*. PHENIX: a comprehensive Python-based system for macromolecular structure solution. *Acta Crystallogr*. *D* **66**, 213–221 (2010)

4. Emsley, P. & Cowtan, K. Coot: model-building tools for molecular graphics. *Acta Crystallogr*. *D* **60**, 2126–2132 (2004)
